# Rapid species-level identification of vaginal and oral lactobacilli using MALDI-TOF MS analysis and 16S rDNA sequencing

**DOI:** 10.1186/s12866-014-0312-5

**Published:** 2014-12-14

**Authors:** Annette Carola Anderson, Mohamed Sanunu, Christian Schneider, Andreas Clad, Lamprini Karygianni, Elmar Hellwig, Ali Al-Ahmad

**Affiliations:** Department of Operative Dentistry and Periodontology, Albert-Ludwigs-University, Hugstetter Strasse 55, Freiburg, Germany; Department of Hygiene and Microbiology, Albert-Ludwigs-University, Hermann-Herder-Str. 11, Freiburg, Germany; Department for Gynaecology, Medical Center, Albert-Ludwigs-University, Hugstetter Strasse 55, Freiburg, Germany

## Abstract

**Background:**

*Lactobacillus* represents a large genus with different implications for the human host. Specific lactobacilli are considered to maintain vaginal health and to protect from urogenital infection. The presence of *Lactobacillus* species in carious lesions on the other hand is associated with progressive caries. Despite their clinical significance, species-level identification of lactobacilli still poses difficulties and mostly involves a combination of different phenotypic and genotypic methods. This study evaluated rapid MALDI-TOF MS analysis of vaginal and oral *Lactobacillus* isolates in comparison to 16S rDNA analysis.

**Results:**

Both methods were used to analyze 77 vaginal and 21 oral *Lactobacillus* isolates. The concordance of both methods was at 96% with five samples discordantly identified. Fifteen different Lactobacillus species were found in the vaginal samples, primarily *L. iners*, *L. crispatus*, *L. jensenii* and *L. gasseri*. In the oral samples 11 different species were identified, mostly *L. salivarius*, *L. gasseri*, *L. rhamnosus* and *L. paracasei*. Overall, the species found belonged to six different phylogenetic groups. For several samples, MALDI-TOF MS analysis only yielded scores indicating genus-level identification. However, in most cases the species found agreed with the 16S rDNA analysis result.

**Conclusion:**

MALDI-TOF MS analysis proved to be a reliable and fast tool to identify lactobacilli to the species level. Even though some results were ambiguous while 16S rDNA sequencing yielded confident species identification, accuracy can be improved by extending reference databases. Thus, mass spectra analysis provides a suitable method to facilitate monitoring clinically relevant *Lactobacillus* species.

## Background

Lactobacilli, gram-positive, highly acidogenic and acid tolerant rods, represent a genus with over 180 known species so far, with lactic acid as their primary fermentation end product [1,2]. The human body hosts various *Lactobacillus* species in different anatomic regions entailing different interactions with the host: e.g. the oral cavity, the intestines and the female genital tract [3-5].

For the latter region lactobacilli play a significant role in the balance of the microbial flora. The healthy female vaginal flora is characterized by different species of lactobacilli and is mostly dominated by one of the following species: *Lactobacillus crispatus, Lactobacillus gasseri, Lactobacillus iners* and *Lactobacillus jensenii* [3,6-10]. Within the last decade, culture-independent studies, involving several ethnic groups, revealed differences in vaginal microbiota as well as the absence of lactobacilli in healthy study participants, particularly Hispanic and black women, complicating the understanding of a healthy versus an unhealthy vaginal flora [10-13]. Despite these recent discoveries, it is still acknowledged that these *Lactobacillus* taxa constitute the predominant genus in the healthy vaginal flora, primarily considering Caucasian women [14-18].

The vaginal lactobacilli play a significant role in protecting their habitat from unwanted invaders. Next to the competition with other species for nutrient supply and adherence to the vaginal epithelium, the production of lactic acid, hydrogen peroxide (H_2_O_2_), bacteriocins and other antimicrobial substances inhibit the vaginal colonization by more pathogenic microorganisms [19]. Consequently, these species help to prevent the shift of the vaginal microbial composition towards high concentrations of mainly anaerobic bacteria such as *Gardnerella vaginalis, Prevotella* spp. or *Atopobium vaginalis* which can lead to a condition known as bacterial vaginosis (BV) [20,21]. Increased bacterial counts for *L. iners* have also been associated with the beginning stages of BV [1,22]. The latter represents the most prevalent vaginal syndrome with a prevalence between 20-50% for fertile, premenopausal women [23], correlating with an increased risk of various infections. Urinary tract infections, sexually transmitted diseases, late miscarriage and preterm birth are the most frequent among these [24-27]. As a result, the maintenance of a healthy vaginal flora is considered very important. To date, many efforts have been made to develop administrable probiotic *Lactobacillus* species to reduce the recurrence of infections and promote a healthy vaginal flora [28,29]. Some of these probiotic lactobacilli have shown promising results in treating and preventing BV as well as preventing urinary tract infections [30-33].

In addition, high counts of *Lactobacillus* species are not only present in the vaginal and intestinal flora, but also in saliva, the supragingival biofilm and advanced carious lesions [34,35]. In the oral biofilm in particular, the bacterial count of *Lactobacillus* species increases when the pH in the oral cavity decreases following carbohydrate metabolism [36].

Caries is characterized by demineralization of tooth enamel in the presence of high concentrations of organic acids that are produced by different species in the oral biofilm [37]. Commonly initial carious lesions develop under the influence of cariogenic bacteria, e.g. mutans streptococci and with the progression of a carious lesion the typical microflora shifts towards more lactobacilli and *Bifidobacterium* spp. as well as other acidogenic and aciduric species [38]. Indeed, various *Lactobacillus* species have been reported in carious lesions [35]. *Lactobacillus fermentum* has been found in childhood caries [39,40] and *Lactobacillus casei/paracasei* as well as *Lactobacillus rhamnosus* have been reported in root caries of elderly patients [41]. Molecular and culture analysis of advanced carious dentine revealed the presence of *Lactobacillus gasseri, Lactobacillus johnsonii, Lactobacillus delbrueckii, Lactobacillus ultunensis, L. crispatus, L. fermentum, Lactobacillus salivarius, Lactobacillus casei/rhamnosus* and other related species [4,42].

Despite the scientific consensus about the significance of the genus *Lactobacillus* for the human host, its species identification still poses several difficulties. Phenotypic detection criteria proved to be unreliable [43], e.g. in distinguishing between *Lactobacillus acidophilus* and closely related species [9,10,44,45]. It was not until molecular identification techniques had been applied that six distinct vaginal *Lactobacillus* species have been identified that had been classified as *L. acidophilus* on the basis of phenotypic characteristics before. Up to now, *L. iners,* as a very common vaginal species, was only described and classified in 1999 with 16S rRNA gene sequencing [14]. *L. iners* can indicate an abnormal flora or the tendency towards BV, e.g. during pregnancy. In general, clear species identification has only been possible using culture-independent methods not based on phenotypic criteria.

Precise species identification is indispensable in light of the findings that different *Lactobacillus* species can have very diverse effects on the host concerning pathogenesis of BV or triggering health-promoting mechanisms, e.g. stabilizing a healthy vaginal flora. Also tools for correct species identification are needed to further elucidate oral pathogenic processes, e.g. the development of advanced caries.

MALDI-TOF-MS analysis is a method which has been used for microbial species identification in routine laboratories for the last decade [46-50]. It holds the potential to provide a fast method for species identification of *Lactobacillus* isolates and would therefore facilitate beneficial monitoring of the vaginal flora. Yet the use of MALDI-TOF MS analysis has not been reported for the species identification of vaginal lactobacilli so far. 16S rDNA-sequencing has been used for routine clinical diagnostics of bacterial species in infectious diseases as well [9,20,51] and provides a highly discriminative comparison method.

To date there is no study comparing MALDI-TOF MS analysis and 16S rDNA-sequencing for the identification of *Lactobacillus* species, isolated from clinical vaginal and oral samples. Therefore this study aimed to assess the capability of MALDI-TOF MS analysis for accurate species identification and the applicability of this method.

## Methods

### Bacterial strains

A total of 77 lactobacilli isolates gained from vaginal samples were used. The vaginal samples were obtained within a gynaecological examination with a speculum using sterile swabs (Sarstedt, Nümbrecht, Germany) that were placed in transport medium. The age range of the subjects was 20–69 (with only 1 subject beyond the reproductive age). All subjects gave their written informed consent to the study protocol. In addition, 21 lactobacilli isolates were collected from supragingival plaque samples and saliva samples. The oral samples were taken from unstimulated saliva and supragingival plaque of healthy male and female volunteers (aged 23–45) that had given their written informed consent to the study protocol. Prior to sampling, a detailed clinical oral examination was performed by a dentist, DMFT values were collected and saliva flow rates were measured at 1.2 ± 0.2. The preconditions for the volunteers were: Healthy dental status, i.e. low DMFT values (decayed, missing, filled teeth) of 0–5, non-smokers, no severe systemic disease, no diseases of the salivary glands, no pregnancy or lactation, no use of antibiotics or local antimicrobial mouth rinses (e.g. Chlorhexidine) within the last month and no participation in another study. The supragingival plaque samples were treated for 30 s in an ultrasonic bath on ice, and dilution series of the samples were prepared as described by Al-Ahmad [52].

Additionally, 10 *Lactobacillus* reference strains from the German Collection of Microorganisms and Cell Cultures (DSMZ, Braunschweig, Germany) were tested.

All bacterial strains and isolates were cultivated on Columbia blood agar plates (CB; Oxoid, Wesel, Germany), Yeast-cysteine blood agar plates (HCB-agar; Becton-Dickinson, Nürnberg, Germany) and MRS agar plates (deMan Rogosa and Sharpe agar; Merck, Darmstadt, Germany). Cultivation was performed aerobically on CB and MRS agar for up to 7 days to capture slow-growing lactobacilli and anaerobically on HCB agar for 3 days (anaerobic chamber, GENbox BioMérieux, Nürtingen, Germany).

### MALDI-TOF sample preparation and MS analysis

Without a detailed extraction step, bacteria from single colonies were used for MALDI-TOF analysis in a MALDI Biotyper Microflex LT (Bruker Daltonik, Bremen, Germany). For the acquisition of the mass spectra the authors followed the manufacturer’s recommendations. The mass spectra were acquired within less than 5 minutes including the sample preparation. The BioTyper 3.0 software compared the obtained spectra with a reference database containing 3740 reference spectra (representing 319 genera and 1,946 species) and expressed the resulting similarity value as a log score. A score of ≥ 2.000 indicated identification on the species level, a score of ≥ 1.700 indicated identification on the genus level whereas any score under 1.700 meant no significant similarity of the obtained spectrum with any database entry. If the results were questionable, the procedure was repeated.

### DNA-extraction, genotypic analysis with 16S rDNA sequencing and phylogenetic analysis

Material of pure cultures was used to extract total bacterial DNA. The colony was placed in 60 μl PBS buffer and 240 μl lysis buffer B (1% Triton X-100 (Sigma-Aldrich, Taufkirchen, Germany), 0.5% Tween 20 (Merck, Darmstadt, Germany), 10 mM TrisHCl (AppliChem, Darmstadt, Germany), 1 mM EDTA (SERVA, Heidelberg, Germany), in ultrapure water (Merck, Darmstadt, Germany), pH 8.0) were added. The solution was heated and incubated at 95 °C for 15 min. Then the lysed cells were centrifuged at 14.000 rpm for 5 min and the supernatant was transferred to a new 1.5 ml vial and used for PCR.

A 1233 bp fragment of the 16S rDNA was amplified using the following primers:

- TP16U1: AGAGTTTGATCMTGGCTCAG and
- RT16U6: ATTGTAGCACGTGTGTNGCCC [53].

The reaction mixture of 50 μl contained 25 μl PCR-Mastermix (Qiagen, Hilden, Germany, including PCR Buffer, deoxyribonucleoside triphosphates and HotStar Taq DNA-Polymerase), 2.5 mM MgCl_2_ (Qiagen, Hilden, Germany) and 300nM of each primer. The PCR cycling conditions consisted of an initial denaturation step at 94°C for 2 min, followed by 30 cycles with denaturation at 92°C for 1 min, annealing at 55 °C at 1 min and extension at 72°C for 1.5 min, with a final extension step at 72°C for 5 min. A no-template control and a positive control were included in each set of PCR reactions. Amplicons were analyzed by electrophoresis on a 1.5% agarose gel and positive reactions were used for sequencing.

The 16S rDNA-PCR products were purified before sequencing, using GFX PCR DNA and Gel Band Purification Kit (GE-Healthcare, Munich, Germany). Subsequently, Cycle Sequencing was performed on a 3130 Genetic Analyzer (Applied Biosystems, Life Technologies GmbH, Darmstadt, Germany) applying the BigDye Terminator Cycle Sequencing Kit (Applied Biosystems, Darmstadt, Germany).

The sequence data obtained was visually proofread and edited and then compared to the public sequence databases, Genbank, EMBL and DDBJ using the BLAST program. The program was run through the server hosted by the National Center for Biotechnology Information NCBI (http://blast.ncbi.nlm.nih.gov/Blast.cgi) [54,55]. Sequences with a ≥ 98% match to a database sequence were considered to be of the same species as the one with the highest similarity and score bits. Additionally, all 16S rDNA sequences were compared with the database RIDOM (Ribosomal Differentiation of Microorganisms, Ridom GmbH, Münster, Germany, accessed through the website www.ridomrdna.de).

The 16 s-rDNA sequences obtained were used for further comparative sequence analysis and phylogenetic analysis using the tools implemented in the software package ARB [56]. To implement the obtained sequences the reference dataset SSURef 111 SILVA NR 98 04 08 12 from the SILVA project [57] was used. Alignments were performed using the SINA Aligner plugin [58]. After manual correction of the alignment, a phylogenetic tree was constructed with the ARB Neighbor joining method applying the Jukes-Cantor correction and with the Maximum Parsimony and Maximum likelihood methods. For the Neighbor joining tree, bootstrapping was calculated based on 1000 replicates. Partial sequences were added without allowing changes of the tree topology by use of the ARB “parsimony interactive” method.

### Data deposition

The partial 16S rDNA sequences supporting the results were deposited in the NCBI Genbank database under the accession numbers KM250382-250423.

The data of the phylogenetic analysis are available from the Dryad Digital Repository: http://doi.org/10.5061/dryad.78vs2.

## Results

### Lactobacilli collection strains

As a preliminary test of the applicability of MALDI TOF mass spectrometry for the identification of *Lactobacillus* species, a set of 10 reference strains were analyzed. The MALDI-TOF MS analysis gave correct results for all strains. The strains and results are listed in Table 1.

**Table 1:** **Reference strains tested with MALDI-TOF MS analysis**

**Species**	Culture collection number*	**MALDI-TOF MS**	**BioTyper log score**
*L. casei*	DSM 20011	L. casei**	2.267
*L. paracasei*	DSM 20020	*L. paracasei*	2.080
*L. fermentum*	DSM 20052	L. fermentum**	1.974
*L. brevis*	DSM 20054	L. brevis**	2.258
*L. delbrückii*	DSM 20074	L. delbrückii**	2.097
*L. plantarum*	DSM 20174	L. plantarum**	2.303
*L. acidophilus*	DSM 20242	*L. acidophilus*	2.337
*L. gasseri*	DSM 20243	L. gasseri**	2.341
*L. salivarius*	DSM 20555	L. salivarius**	2.170
*L. jensenii*	DSM 20557	L. jensenii**	2.089

*DSM: German collection of Microorganisms and Cell Cultures (DSMZ, Braunschweig, Germany).

** Type strains.

### Identification of vaginal and oral isolates with MALDI-TOF MS analysis and 16S rDNA analysis

#### Vaginal isolates

A total of 77 vaginal isolates were tested with MALDI-TOF MS analysis and genotypic analysis using 16S rDNA amplicon sequencing. The isolates belonged to 15 different lactobacilli species and both methods gave concordant results for 75 vaginal isolates, i.e. 97.4%. The most abundant species were *L. iners*, *L. crispatus*, *L. jensenii* and *L. gasseri*, the results are summarized in Table 2. The discordantly or insufficiently identified lactobacilli are listed in Table 3. In addition to 2 of the 5 discordantly identified samples, another 15 samples that gave concordant results with both methods only yielded a MALDI-TOF BioTyper log score of 1.7-1.99, which indicates reliable identification on the genus level but only presumable identification on the species level. However, the resulting species were largely in agreement with the results of the sequencing. For three samples the differentiation between *L. paracasei* and *L. casei* by 16S rDNA analysis was not possible due to the high sequence similarity of both species. Another two samples resulted in sequence data that could not be evaluated.

**Table 2:** **Identification of lactobacilli isolates with MALDI-TOF MS and 16S rDNA-analysis: Vaginal isolates**

**Species**	**16S rDNA-analysis**	**MALDI-TOF-MS**	Concordance [%]*
*L. iners*	18	18	100
*L. crispatus*	15	16	94
*L. jensenii*	14	15	93
*L. gasseri*	13	11	92
*L. fermentum*	4	4	100
L. paracasei**	3	3	100
*L. reuteri*	2	2	100
*L. delbrückii*	1	1	100
*L. mucosae*	1	1	100
*L. harbinensis*	1	1	100
*L. plantarum*	1	1	100
*L. rhamnosus*	1	1	100
*L. sakei*	1	1	100
*L. salivarius*	1	1	100
*L. vaginalis*	1	0	-
Lactobacillus sp.	0	1	-

*Isolates identified as same species by both methods in relation to the total number of isolates of this species identified by 16S rDNA sequencing.

** The 16S rDNA-analysis result was *L. paracasei/L. casei*, both species were not distinguishable from each other.

**Table 3:** **Discordantly identified vaginal and oral isolates**

**Sample Number**	**origin**	**16S rDNA-analysis**	**MALDI-TOF-MS**	**BioTyper log score**
12015	vaginal	*L. gasseri*	*L. crispatus*	1.891*
13015	vaginal	*L. gasseri*	*L. jensenii*	1.944*
12503	vaginal	*L. vaginalis*	Lactobacillus sp*.*	2.276
m113/6spbv1	oral	*L. fermentum*	*L. salivarius*	2.037
m114/10spv15	oral	*L. helveticus*	*L. ultunensis*	1.715*

*log score ≤ 2.0 (and ≥ 1.7) indicates only reliable detection on the genus level, not the species level.

### Oral isolates

A total of 21 oral isolates were tested with MALDI-TOF MS analysis and genotypic analysis using 16S rDNA amplicon sequencing. 19 isolates (90.5%) yielded concordant results with both methods (Table 4). One of the two discordant samples was identified as *L. fermentum* with 16S rDNA analysis but as *L. salivarius* with MALDI-TOF MS analysis, the other one as *Lactobacillus helveticus* with 16S rDNA analysis and *Lactobacillus ultunensis* (BioTyper log score 1.71) with MALDI-TOF MS analysis (Table 3). Altogether three samples yielded BioTyper log scores between 1.85-1.97 which indicates reliable identification only on the genus level.

**Table 4:** **Identification of lactobacilli isolates with MALDI-TOF MS and 16S rDNA-analysis: Oral isolates**

**Species**	**16S rDNA-analysis**	**MALDI-TOF-MS**	Concordance [%]*
*L. salivarius*	3	4	75
*L. gasseri*	3	3	100
*L. rhamnosus*	3	3	100
L. paracasei**	3	3	100
*L. fermentum*	2	1	50
*L. oris*	2	2	100
*L. crispatus*	2	2	100
*L. kalixensis*	1	1	100
*L. reuteri*	1	1	100
*L. helveticus*	1	0	-
L. ultunensis***	0	1	-

* Isolates identified as same species by both methods in relation to the total number of isolates of this species identified by 16S rDNA sequencing.

**The 16S rDNA-analysis result was *L. paracasei/L. casei*, both species were not distinguishable from each other.

***The BioTyper log score was 1.71, reliable identification on genus level, but not on species level, repetition of the samples resulted in *L. acidophilus* with a log score of 1.87.

Overall, the oral isolates represented 11 different species with *L. salivarius*, *L. gasseri*, *L. rhamnosus* and *L. paracasei* identified in more than one sample.

### Comparison of genotypic and MALDI-TOF analysis for vaginal and oral isolates

**Figure 1:**
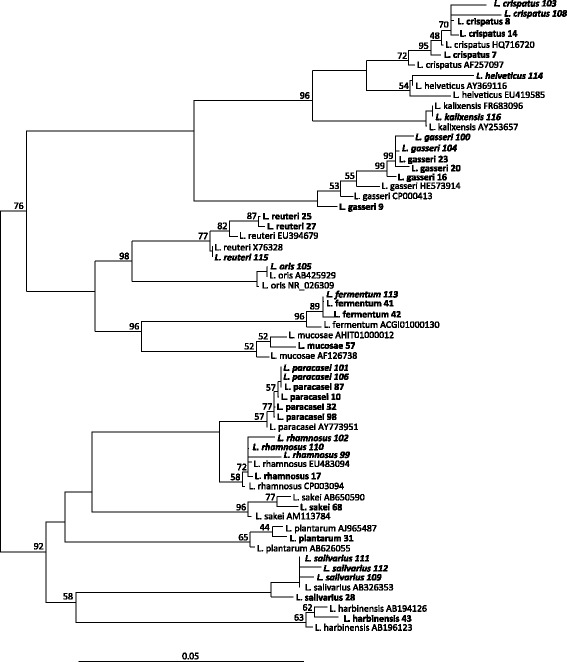
**Phylogenetic tree based on the analysis of 16S rRNA gene sequences derived from oral and vaginal isolates.** 16S rDNA sequences were aligned using the SINA plugin (ARB software package) and distances were calculated using the neighbor-joining method with Jukes-Cantor correction. Reference sequences with accession numbers are in regular type, sample sequences of vaginal isolates in bold type and of oral isolates in bold/italics type. Sample strains are named according to their identification by 16S rDNA sequencing results. Bootstrap values (from 1000 replicates) greater than 40% are shown at the branch points. The bar indicates 5% sequence divergence.

Overall, nineteen different *Lactobacillus* species were identified, the most abundant species found in the vaginal samples were *L. iners*, *L. crispatus*, *L. jensenii* and *L. gasseri*, whereas oral samples (saliva and supragingival plaque) were dominated by *L. salivarius*, *L. gasseri*, *L. rhamnosus* and *L. paracasei*. Altogether 94 out of 98 lactobacilli isolates were analyzed accordantly with both methods, i.e. 96%. The concordance of the methods with reference to the different *Lactobacillus* species and the origin of the isolates is shown in Tables 2 and 4. The detected lactobacilli belonged to six different phylogenetic groups [59], namely the *Lactobacillus acidophilus* group, *Lactobacillus reuteri* group, *Lactobacillus buchneri* group, *Lactobacillus plantarum* group and *Lactobacillus sakei/casei* group. The phylogenetic analysis of 16S rDNA sequences of selected oral and vaginal isolates performed with the ARB software is shown in Figure 1. The phylogenetic analysis showed that oral isolates cluster together and most of them are very close to the vaginal isolates. One oral isolate that was identified as *L. helveticus* with 16S rDNA analysis was identified as *L. ultunensis* / *L. acidophilus* by MALDI TOF MS analysis. In the phylogenetic analysis this sequence clustered with the *L. helveticus* sequences in agreement with the 16S-sequencing result. Analyzed sequences were deposited in the GenBank database.

## Discussion

In this study MALDI-TOF MS analysis was compared with 16S rDNA sequencing to identify 98 lactobacilli isolates from vaginal and oral samples on the species level with the aim of evaluating the potential of mass spectra analysis as a specific and fast detection method. The two methods yielded 96% concordant results. Combining isolates from different clinically relevant sources further affirms the validity of the method. Other studies report similar percentage concordance comparing mass spectrometry with different genotypic methods, e.g. Callaway *et al.* 93% [35] and Angelakis *et al.* 92% [60], or somewhat lower values, e.g. Duskova *et al*. 74% [43] and Dec *et al.* 88.5% [61].

The MALDI-TOF MS analysis demonstrated accurate species-level identification for those *Lactobacillus* species that are of importance for vaginal health as well as those that can indicate the beginning stages of BV. Equally, species associated with progressive caries were identified correctly. Even though MALDI-TOF MS analysis has been used to analyze bacterial species from clinical specimens for nearly two decades [46,62,63] it has not been thoroughly evaluated for vaginal lactobacilli. To date the method has been applied to identify lactobacilli originating from food to the species level [43,60,61,64] and recently attempts have been made to even differentiate subspecies of *Lactobacillus brevis* that are known to cause beer spoilage [65]. Furthermore, Callaway [35] has reported successful species identification of lactobacilli from carious dentine.

16S rDNA sequencing, which was applied as the comparison method, is regarded as an established method for exact species identification and is also applied for clinical diagnostic purposes [66]. Given the known difficulties of exact species identification of lactobacilli by means of phenotypic methods [14,43,67], studies on the taxonomy of lactobacilli or on *Lactobacillus* species identification have been performed using molecular methods based on 16S sequence differences, such as Amplified Ribosomal DNA Restriction Analysis (ARDRA) [67-69], Ribotyping [70,71], 16S rDNA sequencing and others [3,9,18,72]. Identifying lactobacilli employing 16S rDNA sequencing has shown to be a highly discriminative method and has revealed new species, e.g. *L. iners* [73]. Nevertheless, it is not an infallible method and it has failed to differentiate between *L. casei* and *L. paracasei* in our study, due to the substantial similarities of their 16S rDNA genes. In some cases, *Lactobacillus* species can only be correctly identified combining several genotypic methods [61].

Overall, our study demonstrated a high discriminatory power of the mass spectra analysis for the identification of *Lactobacillus* species that are relevant indicators for vaginal health, e.g. *L. crispatus*, *L. gasseri* and *L. jensenii* as well as *L. iners,* a species that is frequently detected in vaginal flora exhibiting a tendency towards BV [1,22]. Before the application of molecular techniques *L. crispatus, L. jensenii* and *L. gasseri*, all belonging to the *L. acidophilus*-group had not been differentiated as distinct species in the past [9]. In our study, all these species were correctly identified by MALDI-TOF MS analysis. Mass spectra analysis proved equally suitable for the detection of *Lactobacillus* species associated with different forms of caries. The species found most frequently in saliva and supragingival plaque samples in this study, namely *L. salivarius*, *L. gasseri*, *L. rhamnosus* and *L. paracasei* concur with those species found in carious lesions [35,72].

Apart from the high accuracy of the method, the rapidity of the analysis is particularly advantageous for the application in clinical laboratories. After obtaining the bacterial isolates the acquisition of the mass spectra, including the sample preparation, takes less than five minutes compared to several hours or even several days by molecular or phenotypic analysis depending on the respective method. Thus the biochemical identification of lactobacilli with the commercial API 50 CHL system (Biomérieux, Marcy l’ Etoile, France) includes fifty reactions and takes an additional 48 h of incubation time after obtaining pure cultures.

Most of the differing results with the two compared methods can be explained: Two samples, identified as *L. gasseri* with 16S rDNA analysis were identified as *L. crispatus* and *L. jensenii* with MALDI-TOF analysis respectively. For both samples, the BioTyper log score was below 2.0, indicating uncertain species identification. Also, *L. gasseri*, *L. jensenii* and *L. crispatus* are all phylogenetically closely related species. The same applies to one oral sample that was identified as *L. helveticus* genotypically and as *L. ultunensis* / *L. acidophilus* by mass spectrometry. Both species are closely related and again the BioTyper log score was below 2.0. The phylogenetic analysis affirms the sequence similarity with *L. helveticus*.

One of the main weaknesses of the mass spectrometry method revealed by our experiments was the frequent occurrence of MALDI-BioTyper log scores between 1.68 and 1.99. Values between 1.7 and 2.0 indicate correct genus identification but only presumptive species identification. Yet from 19 samples showing log scores < 2.0, the vast majority of the presumed species by the BioTyper software (16 samples) agreed with the results achieved by 16S rDNA sequencing, suggesting that despite the low log score the MALDI-TOF MS identification on the species level was correct. This outcome of the mass spectrometry might be improved by extending the BioTyper reference database, as Duskova [43] has stated before. Evidently, the MALDI BioTyper database and corresponding databases of other MALDI-TOF devices will be continuously expanded with the increase of its applications.

## Conclusions

Apart from the above mentioned minor drawback, our study demonstrates the excellent applicability of mass spectra analysis to identify lactobacilli to the species level. The MALDI-TOF MS analysis, e.g. using the commercially available MALDI BioTyper system, provides a fast as well as cost-effective method, which proves to be especially relevant considering the labor intensity of genotypic and phenotypic methods characteristic for *Lactobacillus* species identification. Accurate species identification for clinically relevant species in relation to vaginal health, BV and caries can be achieved in a short time once isolates are obtained. Since it has been shown in recent years that different *Lactobacillus* species have different protective functions for the vaginal flora, specific methods for species-level identification are necessary and profitable for monitoring vaginal health during pregnancy and other stages. MALDI-TOF MS analysis can also facilitate analysis of lactobacilli involved in pathological processes, e. g. progressive caries. Beyond that scope, mass spectra analysis can also help to analyze *Lactobacillus* strains that hold the potential for probiotic application.

### Consent

This study was approved by the Ethics Committee of the Albert Ludwigs-University Freiburg (42/08).
